# Potential Confounder: Bernard and de Burbure Respond

**Published:** 2006-08

**Authors:** Alfred Bernard, Claire de Burbure

**Affiliations:** Unit of Toxicology, School of Public Health, Catholic University of Louvain, Brussels, Belgium, E-mail: bernard@toxi.ucl.ac.be

We thank Aschner for his positive comments and interesting suggestion regarding a possible influence of manganese on serum prolactin levels. We fully acknowledge that the four elements we studied (de Burbure et al. 2006) are probably not the only determinants of serum prolactin levels, especially because variations in blood or urinary levels explained only a few percent of the total variance. Other factors, including perhaps some other metals, most probably also contribute to modulate dopaminergic function and serum prolactin levels. Because the epidemiologic evidence suggesting a link between manganese and serum prolactin in children is recent, we did not measure this metal in our study. However, if we had measured blood manganese in the children in our study, it is far from certain that this factor would have emerged as a significant determinant, possibly confounding the relationships between neurologic markers and blood or urinary levels of cadmium, mercury, and lead.

In the recent study by Wasserman et al. (2006), neurocognitive deficits were indeed not related to blood concentrations of manganese. As to the correlation between blood manganese at birth and cord blood prolactin levels reported by Takser et al. (2004), this association was not adjusted for possible exposure to lead, an indisputable confounder in this sort of study. This being said, we agree of course with Aschner that exposure to manganese should be considered in future studies investigating the developmental effects of heavy metals in the environment.
